# Affective Response Dataset for Virtual Workspaces: Based on Color Stimuli and Multimodal Physiological Signals

**DOI:** 10.3390/s25247461

**Published:** 2025-12-08

**Authors:** Yimeng Zhang, Ting Li, Zihan Li, Jean-Marc Pondo, Xiaobo Wang, Ping An

**Affiliations:** Department of Architecture, School of Architecture and Art, North China University of Technology, Jinyuanzhuang Road 5, Shijingshan District, Beijing 100144, China; litncut@mail.ncut.edu.cn (T.L.); 2024312130135@mail.ncut.edu.cn (Z.L.);

**Keywords:** affective computing, color psychology, virtual reality, physiological signals, workspace

## Abstract

**Highlights:**

**What are the main findings?**

- In terms of emotional valence, the red workspace significantly elicited “anxious”; the yellow space readily induced feelings of “happy”; whereas the blue space was closely associated with a higher sense of “calm”.
- In terms of emotional arousal, the red and yellow workspaces were associated with a higher state of arousal, whereas the blue and green ones resulted in a lower level of arousal. The physiological data solidly supported this subjective arousal reports.

**What are the implications of the main findings?**

- On the theoretical level, this study bridges a gap in the affective dataset of virtual reality workspaces, providing support for the development of “affective-intelligent” virtual workspaces to enhance user experience.
- On the practical level, the distinct emotional impacts of red, yellow, and blue color schemes offer valuable insights for optimizing traditional workspace designs, thereby contributing to the improvement of the participants’ mental health.

**Abstract:**

In the context of post-pandemic remote work normalization and the emergence of the metaverse, virtual workspaces have attracted significant attention as critical digital infrastructure with promising application prospects. While virtual workspaces enable efficient task performance, compared with traditional ones, the lack of emotional connection between humans and machines adversely affects participants’ mental health. The emergence of affective computing has made it possible to endow virtual workspaces with “affective intelligence”. Therefore, this study aims to clarify the relationship between color and participants’ emotions in virtual workspaces through an experiment involving 48 participants, and eight virtual workspaces were constructed, incorporating four color conditions (red, blue, yellow, and green) and two workspace types (shared and single). Data were synchronously collected using the Positive and Negative Affect Schedule (PANAS), a questionnaire item on arousal, electrodermal activity (EDA), and heart rate variability (HRV). The results successfully established specific associations between colors and emotions: red with “anxious”, yellow with “happy”, and blue with “calm”. Although no specific emotion word was identified for green, this study successfully achieved the emotion classification of virtual workspaces and constructed a corresponding dataset. These findings provide a theoretical foundation for the development of affective computing models.

## 1. Introduction

Since the outbreak of the COVID-19 pandemic, remote work models have become widespread and increasingly normalized, especially in better-educated and higher-paid sectors [1]. From 2019 to early 2023, job postings offering remote work increased more than threefold in the U.S. and up to fivefold in other English-speaking countries [2]. With the rise of the metaverse concept, virtual workspaces, as a critical component of future digital infrastructure, are attracting growing attention for their promising application prospects. However, compared to traditional physical work environments, existing virtual workspaces, while supporting efficient collaboration, generally lack effective support for human–computer emotional interaction. This deficiency makes it difficult to establish deep emotional connections, potentially exerting negative psychological effects on participants, such as emotional exhaustion, stress, anxiety, and reduced job satisfaction, ultimately affecting the mental health and overall well-being of remote workers [3]. Therefore, constructing “affective intelligence” virtual work environments capable of real-time perception, recognition, and adaptation to participants’ emotional states has become a crucial challenge in enhancing future work efficiency and participants’ psychological well-being.

Immersive VR systems enhance social presence and collaborative efficiency by generating shared, embodied 3D spaces. Furthermore, the high controllability and immersive nature of VR make it an ideal platform for investigating and intervening in the psychological impacts of virtual work, such as emotional stress. Affective computing aims to enable systems to recognize, interpret, and respond to human emotions [4]. Building on this premise, our research leverages VR’s capability to dynamically modify environmental parameters (e.g., color) and integrates it with affective computing, thereby providing a viable pathway toward constructing “emotionally intelligent” virtual work environments. A critical first step towards such intelligent systems is to establish a foundational understanding of how specific design elements within virtual environments influence occupant affect. As a primary visual factor, environmental color is known to significantly shape emotional experiences [5], yet its systematic investigation within the context of virtual workspaces remains limited.

To bridge this gap, this study aimed to systematically investigate the correspondence between color schemes and participants’ emotions within immersive virtual workspace scenarios. By employing a multimodal approach that synchronizes physiological data with self-reported measures, this research seeks to create a dedicated affective dataset for virtual workspaces. The findings are expected to provide a crucial empirical and data-driven foundation for the future development of adaptive affective computing models, ultimately contributing to the construction of more humanized and psychologically supportive virtual work environments.

## 2. Related Works

### 2.1. Affective Computing for Emotion Recognition

Affective Computing (AfC), as a significant branch of artificial intelligence, was first proposed by Rosalind Picard in 1997. It aims to develop computational systems capable of automatic recognizing, modeling, and responding to human emotions through interdisciplinary approaches involving fields such as psychology, computer science, and biomedical engineering [6]. This technology has demonstrated broad application potential across various domains, including education and healthcare [7].

Affective computing involves two key processes: emotion classification and emotion elicitation. The field of emotion classification has commonly employed two principal approaches: the discrete model and the dimensional model. According to the discrete model, complex emotions are formed through the combination of a small set of basic emotions. For example, Ekman [8] proposed six basic emotions, including anger, disgust, fear, joy, sadness and surprise. The second is the dimensional model. For instance, Russell and Mehrabian proposed the “Circumplex Model of Affects” (CMA) [9], which conceptualizes emotions along the two primary dimensions of valence, i.e., the degree to which an emotion is perceived as positive or negative; and arousal, i.e., how strongly the emotion is felt. By positioning valence and arousal on the axes of a Cartesian coordinate system, any emotion can be defined within this conceptual space [10].

For automatic emotion classification, researchers have extensively utilized multimodal data including vocal characteristics, facial expressions, body posture, and physiological signals from participants [11]. For stress-prone workspaces, previous studies have utilized wearable sensors to measure color, including heart rate (HR) [12], heart rate variability (HRV) [13,14], and electrodermal activity (EDA) [15]. The integration of multiple machine learning models has enabled highly accurate stress detection. However, while these studies provide valuable resources for recognizing stress, they typically focus on detecting pre-existing stress states induced by general work demands, rather than systematically investigating how specific, controllable design parameters of the workspace can be manipulated to elicit emotional states.

### 2.2. VR for Emotional Elicitation and Environmental Design

Substantial research has confirmed that immersive virtual environments (IVEs) can elicit emotion, cognitive, and sensory responses from participants that are highly consistent with those evoked in real-world settings, thereby serving as effective experimental substitutes for physical scenarios [16,17,18]. Specifically, Maffei et al. [18] demonstrated no significant differences in overall perceptual quality (e.g., visual and auditory) between the two environments; Peñate et al. [17] provided neuroscientific evidence that virtual stimuli can induce emotional responses comparable to those triggered by real stimuli; and in the context of office environments, Latini et al. [16] further found no significant divergence in the participants’ evaluations of comfort, productivity, and behavior between real and immersive virtual settings.

Regarding emotion elicitation, while classic datasets in emotion elicitation primarily use images, audio, and video as stimuli [19,20], employing virtual reality (VR) scenarios can elicit a more potent emotional response compared to video stimuli alone [21]. Previous studies have established emotion datasets or proposed novel emotion recognition models by employing interactive 3D virtual environments [22] or VR technology [4,23].

As a critical visual element in environmental design, color exerts a significant influence on individuals’ emotions, cognition, and behavior. Previous studies have demonstrated that different colors elicit distinct emotional responses; for instance, red is often associated with negative emotions such as tension and unpleasantness [5,24], yellow is most frequently linked to “happiness” [24], while blue and green are more likely to induce feelings of calmness [25]. Furthermore, research has found that red evokes a stronger arousing effect than colors such as green, blue, or yellow [26,27,28].

This potential is exemplified in studies that have employed VR to study color–emotion interactions. Although previous research has attempted to investigate the impact of work environmental color on emotions using Virtual Reality (VR) technology [5], most studies have merely employed VR as a tool to enhance ecological validity. Notable exceptions include the work of Marín-Morales et al. [4], who employed four VR indoor environments with varying colors and materials to elicit emotions, simultaneously collecting participant data on electroencephalogram (EEG), electrocardiographic (ECG), and self-reports. They successfully achieved a four-category emotion classification and developed an accurate emotion recognition model by integrating a Support Vector Machine (SVM) machine learning approach, thereby validating the effectiveness of immersive virtual environments in both eliciting and automatically identifying distinct emotional states. Similarly, Dozio et al. [22] developed and validated a database of interactive 3D virtual environments for emotion elicitation by analyzing self-reported emotion ratings from 75 participants. While these studies validate the feasibility of using affective computing within immersive virtual environments, it is noteworthy that they were not conducted within the specific context of virtual workspaces, which possess unique functional and emotional requirements.

### 2.3. Research Gap and Objectives

The related works revealed significant gaps that this study aims to address. First, while previous research has established the general emotional impact of environmental colors and validated VR’s efficacy in emotion elicitation, systematic investigations specifically examining color–emotion relationships within virtual workspace environments remain limited. Second, despite the proven capability of multimodal physiological data for emotion recognition, its application to understanding how virtual workspace design parameters influence participants’ affective states represents an underdeveloped research direction. Third, and most fundamentally, there is a notable scarcity of dedicated affective datasets that quantitatively link workspace design elements with participants’ multimodal emotional responses, which constitutes a major barrier to developing data-driven affective computing frameworks for virtual workspaces.

To address these research gaps, this study established the following objectives:

(1) To empirically investigate the correspondence between color schemes and participants’ emotions through controlled virtual workspace scenarios, employing synchronized multimodal data collection incorporating physiological measurements and subjective reports.
(2) To construct a comprehensive Virtual Workspace Affective dataset that captures the relationship between environmental color conditions and emotional responses, thereby addressing the critical data scarcity in virtual workspace contexts.
(3) To establish an empirical foundation for emotion–color associations in virtual workspaces, providing both the essential dataset and methodological framework necessary for future development of adaptive affective computing systems.

Through these objectives, this research aims to bridge the current gap between affective computing capabilities and virtual workspace design by establishing a solid empirical foundation for developing intelligent virtual environments that can effectively respond to participants’ emotional needs.

## 3. Materials and Methods

### 3.1. Experimental Environment

**Figure 1:**
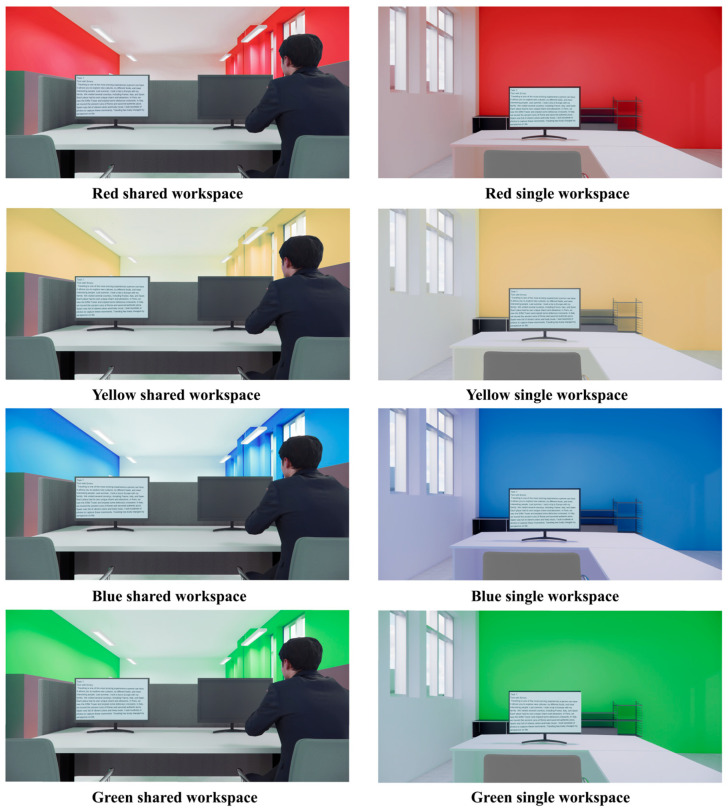
Experimental scenes.

The study employed immersive virtual reality (VR) to simulate different workspace scenarios, which served as visual stimuli to elicit the participants’ emotional responses. Based on existing studies regarding color–emotion associations, red, yellow, blue, and green were selected as the crucial visual variables for this experiment, as they have been demonstrated to evoke a broad spectrum of emotions. Additionally, two prevalent workspace types, “shared workspace” and “single workspace”, were included as a second experimental factor. These workspace conditions were crossed with the color variations to create the full set of experimental conditions (Figure 1).

The virtual workspaces in this study were constructed using the BIM software Revit (Version 20230308_1635, Autodesk, Inc., San Rafael, CA, USA) for scene modeling, while the Enscape (Version 4.1.0 Preview3, Enscape GmbH, Karlsruhe, Germany) software was employed for VR scenes rendering and panoramic image export. The exported panoramic images were subsequently processed and edited using the specialized software Pano2VR (Version 7.1.6.20966, Garden Gnome Software, Vancouver, BC, Canada). These processed images were then displayed via Steam VR (Version 01.00.00.02, Valve Corporation, Bellevue, WA, USA) and ultimately presented to the subjects through a portable head-mounted display (HMD) (HTC VIVE Pro 2, HTC Corporation, Taiwan, China). This immersive virtual environment was effective in eliciting emotional states, and even subtle changes within the environment can induce physiological responses from users [29]. Furthermore, the 360° panoramic format provided by HMD serves as an effective setup for evoking psychological and physiological responses comparable to those evoked in real-world environments [30].

The virtual workspace employed a hybrid lighting scheme, integrating stable artificial light sources with comfortable and soft natural illumination. The system maintained an illuminance level of 500 lux and a correlated color temperature (CCT) of 4500 K. The 500 lux illuminance ensures sufficient and uniform light distribution across work surfaces, while the 4500 K color temperature contributes to a focused and comfortable working atmosphere. This parameter combination is in full compliance with established lighting standards and guidelines for workspace environments. Furthermore, to guarantee the accuracy and reliability of experimental data, the research team implemented rigorous control over all lighting parameters throughout the entire duration of the study.

To create a more immersive workspace and enhance ecological validity, this study configured the immersive virtual reality workspace environment with furniture matching that of a real workspace. Furthermore, colleague avatars were incorporated into the immersive virtual shared workspace scenario to simulate real-world working conditions more authentically. As participants were required to remain seated throughout the experiment, the virtual seat height was calibrated to match the physical laboratory chair height, thereby enhancing situational presence.

### 3.2. Participants

The research recruited a total of 48 healthy volunteers aged 18 to 34, with the participant pool primarily consisting of undergraduate and graduate students. In addition to Chinese students, international students were also included. Their demographic information is summarized in Table 1.

This study utilized G*Power 3.1.9.7 [31] to perform a power analysis. A repeated measures ANOVA (within factors) model was employed, incorporating four color conditions and two workspace type conditions, with the color variable measured eight times repeatedly. With α = 0.05 and a statistical power target of 0.80, and assuming a correlation of 0.50 among repeated measures, we estimated the required sample sizes based on Cohen’s effect size criteria [32]: a small effect size (f = 0.10) required 91 participants, a medium effect size (f = 0.25) required 16 participants, and a large effect size (f = 0.40) required only 7 participants. Considering that effect sizes reported in this research field generally fall within the medium range, a final sample size of 48 participants was determined. This sample size is considered sufficient to detect medium or larger effects. Therefore, the study meets the required sample size criteria.

### 3.3. Questionnaires

The emotional states of participants were assessed along the dimensions of valence and arousal. Valence was measured using the Positive and Negative Affect Schedule (PANAS), selecting six adjectives potentially relevant to the workspace from ten pairs: “happy”, “excited”, “calm”, “annoyed”, “anxious”, and “sad” [33]. Arousal was evaluated via a single-item question: “Based on your current physical feelings, please indicate your level of agitation or calmness”. To evaluate the ecological validity of the virtual workspace, the Igroup Presence Questionnaires (IPQ) was employed to measure the sense of presence across its four dimensions: general presence (GP), spatial presence (SP), involvement (INV), and realism (REAL) [34,35]. Furthermore, the Virtual Reality Sickness Questionnaire (VRSQ) was administered to quantify cybersickness through the severity of nine symptoms: nausea, dizziness, headache, eyestrain, fatigue, difficulty focusing, blurred vision, general discomfort, and difficulty concentrating [36]. All of the above questionnaires employed a five-point Likert scale for scoring.

Reliability analysis was conducted utilizing Cronbach’s alpha (α) to examine the internal consistency of the PANAS, IPQ, and VRSQ questionnaires. The analysis yielded Cronbach’s alpha coefficients of 0.802 for the PANAS, 0.897 for the IPQ, and 0.804 for the VRSQ. Since these values exceeded the established threshold of 0.70–0.80 [37], the questionnaire items were deemed to possess high reliability.

### 3.4. Physiological Measures

Electrodermal activity (EDA) (KiNGFAR INTERNATIONAL Inc., Beijing, China) and photoplethysmography (PPG) signals (KiNGFAR INTERNATIONAL Inc., Beijing, China) were recorded and analyzed using an Ergo LAB wearable wrist sensor. The EDA is closely associated with an individual’s emotions, arousal, and attention, comprising two primary components: the tonic and phasic data. Tonic data refer to the slowly varying signals in the overall physiological record, typically measured as skin conductance level (SCL); whereas phasic data refer to the rapidly changing components elicited by sympathetic nervous activity, also identified as Skin Conductance Responses (SCRs), which are characterized by more rapid fluctuations and larger amplitudes. In line with previous research, SCL was selected as the indicator for emotional arousal [38,39]. Furthermore, a time series of inter-beat intervals was extracted from the PPG pulse signal, from which heart rate variability (HRV) metrics were derived by converting the series into inter-beat interval (IBI) data points. Based on previous research, the following indices were chosen to represent arousal levels: heart rate (HR) [5], Standard Deviation of Normal-to-Normal intervals (SDNN) [40], Root Mean Square of Successive Differences (RMSSD) [41], HRV-nLF [42], and HRV-nHF [42]. HRV-nLF and HRV-nHF are frequency-domain metrics of HRV. Specifically, HRV-nLF represents the low-frequency band of signal (LF, 0.04–0.15 Hz) in normalized units, which is associated with sympathetic nervous activity and increased arousal; whereas HRV-nHF corresponds to the high-frequency band of signal (HF, 0.15–0.4 Hz) in normalized units, which is linked to parasympathetic nervous activity and decreased arousal [43,44].

### 3.5. Experimental Procedure

**Figure 2:**
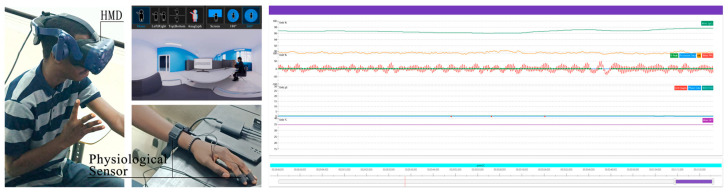
Devices used for the experiment.

**Figure 3:**
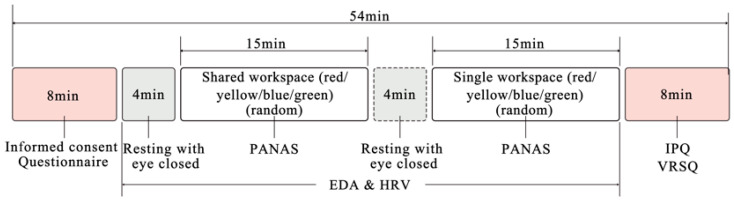
Experimental procedure. Note: PANAS (Positive and Negative Affect Schedule); EDA (electrodermal activity); HRV (heart rate variability); IPQ (Igroup Presence Questionnaires); VRSQ (Virtual Reality Sickness Questionnaire).

Prior to the experiment, participants were required to sign an informed consent and complete a background questionnaire, followed by reviewing the experimental information. The researcher provided a detailed explanation of the rating scales to ensure full comprehension before the experiment commenced. The entire experiment lasted 54 min, including a 4 min eyes-closed baseline period after the start. All data were processed anonymously. During the experiment, participants were required to remain physically still, with only head movement permitted, and to report their emotions in real-time using the 5-point PANAS scale, while their physiological data were simultaneously recorded. It is noteworthy that participants provided all self-reported feedback via manual gestures rather than verbal responses. This method was chosen to standardize reporting across participants and, critically, to prevent speech-induced cognitive load and muscle artifacts from contaminating the concurrent physiological data, such as electrodermal activity [45]. The experiment comprised two sets of VR panoramic scenes: shared workspace and single workspace, with four scenes in each set. The order of the two sets was fixed (shared followed by single). However, the scenes within each set were presented in a randomized order to prevent sequence effects and biases that could arise from a fixed or balanced presentation. Each scene was viewed for 3 min, followed by a 1 min rest interval. This was followed by a mandatory 1 min rest interval to allow physiological arousal and subjective emotional state to return to baseline, thereby preventing carry-over effects and ensuring that each trial’s data reflect its specific stimulus. A 4 min eyes-closed rest period was provided between the two sets. Upon completion, participants filled out the IPQ and the VRSQ. A researcher was present throughout the entire process to provide assistance (Figure 2 and Figure 3).

### 3.6. Statistical Analysis

This experiment employed a three-factor mixed design. The independent variables primarily included color and workspace type. Given that the participants came from different cultural backgrounds, cultural background was also included as a variable. The dependent variables involved data from PANAS vocabulary, PA, NA, SCL, HR, SDNN, RMSSD, HRV-nLF, and HRV-nHF. The total scores for PA and NA were calculated based on the PANAS.

Since the PANAS vocabulary is not a continuous variable, and NA, SCL, SDNN, and RMSSD violated the assumption of normality, along with the residuals for HR data being non-normal, the traditional three-factor mixed ANOVA was deemed unsuitable for these measures. Consequently, the Aligned Rank Transform ANOVA (ART-ANOVA) was applied to analyze these variables. For the variables PA, HRV-nLF, and HRV-nHF, which met the assumptions of sphericity, homogeneity of variances, and normally distributed residuals, the traditional three-factor mixed ANOVA was utilized.

The ART-ANOVA was conducted using R version 4.2.3, while the traditional three-factor mixed ANOVA was performed using SPSS version 23.0.0.0. For the ART-ANOVA, post hoc pairwise comparisons for main effects employed Holm–Bonferroni correction for *p*-values. For the traditional three-factor repeated measures ANOVA, post hoc pairwise comparisons for main effects utilized Bonferroni correction.

For the analysis of effect sizes, the magnitudes of these effects were interpreted in accordance with Cohen’s conventional thresholds [32]: specifically, for |d|, values < 0.20 were considered small, 0.20 ≤ |d| < 0.50 medium, and |d| ≥ 0.80 large. Effect sizes based on partial eta squared (η_p_^2^) were conventionally interpreted as negligible (η_p_^2^ < 0.04), small (η_p_^2^ ≥ 0.04), medium (η_p_^2^ ≥ 0.25), or large (η_p_^2^ ≥ 0.64) [46].

## 4. Results

### 4.1. PANAS

The statistical analysis of the Positive and Negative Affect Schedule (PANAS) data revealed a compelling and nuanced picture of how different colored environments influence emotions. The results confirmed that there were statistically significant differences in the reported intensity for each of the six measured emotions across the four colors (red, yellow, blue, and green).

The specific details are as follows:

- Happy: F (3, 138) = 15.678, *p* < 0.001, η_p_^2^ = 0.287.
- Excited: F (3, 138) = 3.588, *p* = 0.015, η_p_^2^ = 0.072.
- Calm: F (3, 138) = 19.392, *p* < 0.001, η_p_^2^ = 0.297
- Annoyed: F (3, 138) = 23.341, *p* < 0.001, η_p_^2^ = 0.337.
- Anxious: F (3, 138) = 14.173, *p* < 0.001, η_p_^2^ = 0.236.
- Sad: F (3, 138) = 5.823, *p* < 0.001, η_p_^2^ = 0.112
- Table 2 presents the results for each emotion label when it was rated highest in its corresponding color, compared against all other colors (e.g., participants’ rates for the “happy” label in the yellow workspace compared to the rates for the same label in all the other color conditions).

**Happy.** A distinct and statistically significant association was observed between a yellow workspace and the “happy” mood. Specifically, happiness ratings in the red (M = 2.646; estimate = −80.889; t (138) = −4.800; *p* < 0.001; Cohen’s d = −0.634), blue (M = 2.677; estimate = −79.083; t (138) = −4.693; *p* < 0.001; Cohen’s d = −0.588), and green (M = 2.729; estimate = −81.056; t (138) = −4.810; *p* < 0.001; Cohen’s d = −0.616) conditions were significantly lower than those in the yellow condition (M = 3.448). The statistical results indicated that all pairwise comparisons reached a high level of significance, demonstrating that the yellow environment possesses a unique capacity for eliciting feelings of happiness. This finding suggests that incorporating the color yellow into workspaces could be an effective strategy for enhancing emotional well-being.

**Calm.** The emotion of calm was most strongly associated with the blue workspace, which achieved a mean rating of 3.365. The post hoc analysis revealed that this sense of calm in the blue workspace was significantly more intense than in the red condition (M = 2.031; estimate = 92.785; t (138) = 5.186; *p* < 0.001; Cohen’s d = 1.088) and significantly greater than in the green condition (M = 2.760; estimate = 44.569; t (138) = 2.491; *p* = 0.028; Cohen’s d = 0.486). A particularly interesting finding emerged from the comparison between blue and yellow. While yellow (M = 3.385) was the primary catalyst for happiness, it also fostered a level of calm that was statistically indistinguishable from that in the blue environment (*p* = 0.513). This suggests that yellow, in this study, possessed a dual character, promoting both happiness and calmness to a similar degree as blue, which was a more specialized elicitor of calm.

**Anxious.** Conversely, the red workspace was predominantly linked to the negative, high-arousal emotion of anxious. Participants reported significantly higher anxiety in the red condition (M = 2.656) than in the yellow (M = 1.500; estimate = 102.257; t (138) = 5.602; *p* < 0.001; Cohen’s d = 1.013), blue (M = 1.823; estimate = 63.431; t (138) = 3.475; *p* = 0.003; Cohen’s d = 0.697) workspaces, and green (M = 1.969; estimate = 54.632; t (138) = 2.993; *p* = 0.013; Cohen’s d = 0.554) workspaces. This consistent pattern across all comparisons positions red as a potent trigger for anxiety relative to the other colors tested.

In summary, the analysis revealed a clear and distinct pattern of color–emotion associations. The red workspace was unequivocally identified as a primary elicitor of “Anxious” feelings. Conversely, the yellow workspace predominantly elicited “happy” emotions, positioning it as a potent stimulant of positive states. The blue workspace was most strongly linked to high “calm” ratings, affirming its well-documented role in promoting tranquility positive affect. However, no single emotion term emerged as being uniquely and predominantly elicited by the green workspace within the parameters of this study. This suggests that green’s emotional impact may be more nuanced and context-dependent.

### 4.2. Arousal

The threshold for color-induced emotional arousal was calculated by first aggregating the self-reported arousal ratings from all participants across the four-color conditions (red, yellow, blue, and green). The median of these ratings was then computed, yielding a value of 2.500. This value corresponds to the “midpoint” of the arousal level within the participants and serves as a benchmark for distinguishing between “relatively high arousal” and “relatively low arousal”.

Subsequently, to characterize the typical arousal response to each color, the mean arousal rating was computed alongside its standard deviation. Data are presented as Mean ± SD, concisely summarizing the tendency of arousal levels for each workspace. As detailed in the statistical results presented in Table 3, the application of this classification scheme revealed a clear division among the color stimuli. The red and yellow workspaces both yielded mean arousal scores that were conclusively above the 2.500 threshold, thereby categorizing them as high-arousal environments. This finding is consistent with color psychology theories that often associate these wavelengths with energy and stimulation [47]. In contrast, the blue and green workspaces produced mean scores below the threshold, leading to their classification as low-arousal environments. This aligns with their common perceptual associations with calmness and peacefulness [48]. Thus, using this threshold, the four colors were divided into two distinct categories based on their level of arousal.

### 4.3. Physiological Data

The statistical analysis of the physiological data yielded a clear and consistent pattern across the four colors. Specifically, for the key indicators of autonomic nervous system activity—including skin conductance level (SCL) and the heart rate variability indices of HR, SDNN, RMSSD, HRV-nLF, and HRV-nHF—the results of the test showed no statistically significant differences. This conclusion is supported by the obtained *p*-values: SCL (*p* = 0.411), HR (*p* = 0.586), SDNN (*p* = 0.425), RMSSD (*p* = 0.793), HRV-nLF (*p* = 0.615), and HRV-nHF (*p* = 0.404), all of which were substantially greater than the conventional alpha level of 0.05.

**Figure 4:**
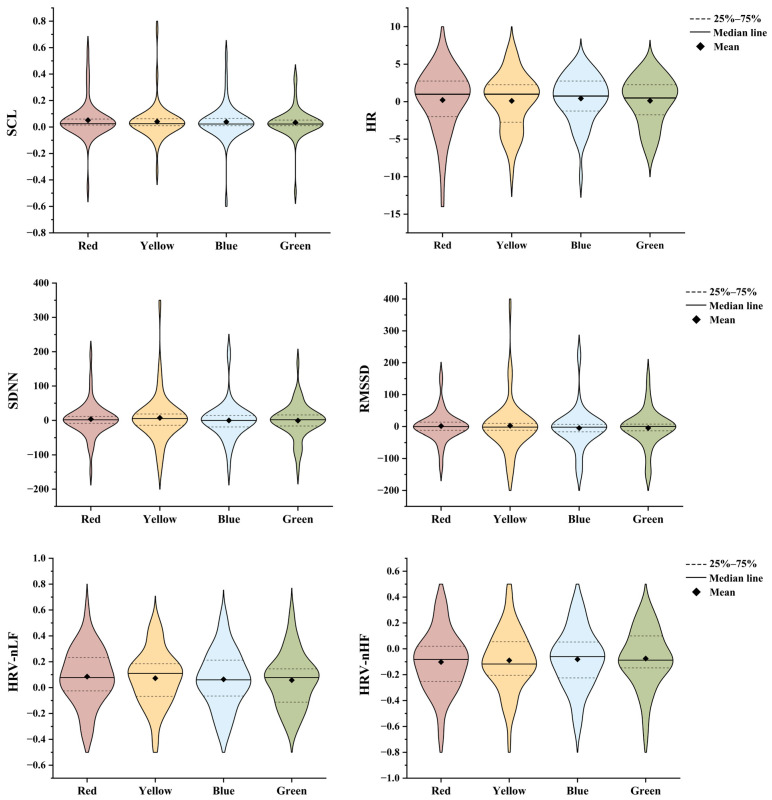
Statistical results of physiological data.

The HR data used in the study were difference scores calculated by subtracting baseline values. Although no statistically significant differences in HR were found among the four colors, the descriptive data (i.e., mean values above zero for all colors) suggest that physiological arousal in the virtual workspace may have been higher than at baseline. Furthermore, as shown in Figure 4 metrics including SCL, SDNN, and RMSSD did not exhibit clear trends across the four-color conditions.

While the statistical analysis confirmed the absence of statistically significant differences in the HRV-nLF and HRV-nHF across the four colored workspaces, a nuanced examination of the descriptive data revealed a physiologically coherent and theoretically meaningful pattern. The data visualized in Figure 4 presented a clear change in both HRV-nLF and HRV-nHF values across the color from red to green.

HRV-nLF is often interpreted as an indicator of sympathetic nervous system (SNS) activity and increased arousal [44]. The mean values demonstrated a progressive decrease across the color conditions. The highest HRV-nLF value was recorded in the red workspace (M = 0.085), followed by a decline in the yellow workspace (M = 0.072). This decreasing trend continued through the blue workspace (M = 0.064) and reached its lowest point in the green workspace (M = 0.058). Although not statistically significant, this declining trend is visually consistent with a potential decrease in sympathetic drive as the environmental color shifts from red to green.

Conversely, for HRV-nHF, a metric predominantly linked to parasympathetic nervous system (PNS), vagal activity, and reduced arousal [44], the data exhibited a mirroring, gradual increase across the same sequence. The lowest HRV-nHF value was observed in the red workspace (M = −0.102). A slight increase was noted in the yellow workspace (M = −0.090), followed by a further rise in the blue workspace (M = −0.081), culminating in the highest HRV-nHF value within the green workspace (M = −0.075). The observed trend in the mean values coincides with the interpretation that parasympathetic activity may increase from red to green environments; however, this interpretation remains speculative due to the lack of statistical significance.

In summary, the observed descriptive trends—specifically, a decrease in SNS-associated HRV-nLF means and an increase in PNS-associated HRV-nHF means from red to green—while not statistically significant, are directionally consistent with established physiological principles. Consequently, these preliminary patterns suggest a hypothesis for future research: physiological arousal may progressively decrease when transitioning from a red workspace environment to yellow, then to blue, and finally to green. This potential relationship requires validation in larger-scale studies.

### 4.4. Effects of Cultural Background and Workspace Type on Emotion

The results of the three-factor mixed ANOVA revealed that for PA, the main effect of workspace type was not significant. However, the main effect of cultural background was significant [F (1, 46) = 17.866, *p* < 0.001, η_p_^2^ = 0.28]. Post hoc comparisons (with Bonferroni correction) indicated that African participants reported significantly higher (M = 10.396) PA than Asian participants (M = 7.646), with a mean difference of 2.750, 95% CI [1.441, 4.059], *p* < 0.001. Furthermore, the color × cultural background interaction, the workspace type × color interaction, and the cultural background × color × workspace type interactions were all non-significant.

Since all *p*-values exceeded 0.05, there were no significant main effects of cultural background or workspace type, nor any significant cultural background × color interaction, workspace type × color interaction, and cultural background × color × workspace type interaction effects were found for any of the measures of, NA, SCL, HR, SDNN, RMSSD, HRV-nLF, or HRV-nHF.

### 4.5. IPQ and VRSQ

The IPQ scores obtained in this study were: GP = 3.25, SP = 3.76, INV = 3.63, and REAL = 3.81. Thus, all dimensions were above the moderate level (i.e., above 3 on the 5-point scale), suggesting that the participants generally experienced a reasonable sense of presence. Specifically, the scores corresponded to a participant experience characterized by a strong sense of location within the environment within the environment (SP), active involvement in VR tasks (INV), and a perception of its realism (REAL).

The VRSQ results showed that 91–95% of participants reported no or slight symptoms of “nausea”, “headache”, and “eyestrain”, suggesting that the immersive VR experience used in this study was well-tolerated and comfortable for most participants. Other symptoms, including “dizziness”, “difficulty concentrating”, and “general discomfort”, could be considered non-significant, as 83–89% of participants rated these as “0 (none)” or “+1 (slight)”. On the other hand, at least 25% of participants reported noticeable symptoms (rated “+3” or “+4”) in “difficulty focusing”, which may be associated with “blurred vision” and “fatigue.”

In summary, the immersive VR experience effectively induced a sense of presence in participants while maintaining controllable side effects, thereby confirming the ecological validity of the virtual workspace and supporting the suitability of VR color as a form of stimulus.

## 5. Discussion

This study systematically investigated emotional responses within an immersive virtual workspace environment, employing four color schemes and two spatial types as carefully designed VR stimuli. A comprehensive dataset was compiled from 48 participants through synchronized collection of both subjective self-report measures and objective physiological signals. The subjective assessment included the Positive and Negative Affect Schedule (PANAS) and arousal ratings, while physiological monitoring captured electrodermal activity (EDA) and heart rate variability (HRV) parameters. Through data analysis, the successful classification of emotions within the virtual workspace was achieved, and a corresponding dataset was constructed. Furthermore, the findings of this study provide a theoretical foundation for developing affective computing models for virtual workspaces.

Regarding the ability of workspace colors to elicit specific emotions, the study analyzed the data through the emotional dimension models, focusing on valence and arousal.

Firstly, in terms of emotional valence, the results from the Positive and Negative Affect Schedule (PANAS) provided clear differentiations. The red workspace was primarily characterized by the elicitation of “anxious” feelings. This outcome is highly consistent with the work of Sutton and Altarriba [24], which identified red as most frequently associated with negative emotions. In addition, research found that the yellow workspace predominantly elicited the emotion “happy”, and the blue space was associated with a higher incidence of “calm”. This finding is consistent with previous research linking blue to “calmness” [25], as well as with a study identifying yellow as the color most frequently associated with “happiness” [24]. No specific emotion word was found to be uniquely associated with the color green, which contradicts previous research indicating that green is more likely to induce feelings of calmness [25]. A subsequent analysis specifically targeting the term “calm” revealed that green (M = 2.760) was rated as significantly more calming than red (M = 2.031; estimate = 48.215; t (138) = 2.695; *p* = 0.024; Cohen’s d = 0.590), and was perceived as less calming than both blue (M = 3.365; estimate = −44.569; t (138) = −2.491; *p* = 0.028 Cohen’s d = −0.486) and yellow (M = 3.385; estimate = −56.313; t (138) = −3.147; *p* = 0.008; Cohen’s d = −0.481). This discrepancy suggests that the emotional effects of colors may not be inherent or absolute properties, but rather are subject to the complex influence of a range of moderating variables. Given the diverse cultural backgrounds of the participants in this experiment, cultural context may have influenced the results.

Furthermore, the present study presented multiple colors—including blue, green, yellow, and red—simultaneously. Under such direct comparison, the “calm” quality of blue was accentuated to the greatest extent, making it the most tranquil color. Meanwhile, the “brightness” and “warmth” conveyed by yellow may have been interpreted by participants as a soft, comfortable, and positive form of calmness, rather than as high-arousal excitement. Green, in this context, occupied an intermediate position—it was not as aggressive or high-arousal as red, yet it did not surpass the pure calmness associated with blue or, in certain cases, yellow.

Secondly, the analysis of arousal levels further refined the emotional states of each color. The study found that the emotions elicited by both red and yellow were of a high-arousal level. For red, this high arousal manifested as the tense alertness of anxiety, while for yellow, it presents as the lively vitality of happiness. On the other end of the spectrum, the emotions elicited by blue and green were classified as low arousal. The calmness associated with blue is inherently tranquil, and while the study did not pinpoint a single dominant emotion word for green, its placement in the low-arousal category suggests it promotes a state of relaxation and quietude. The subsequent analysis of physiological data aimed to provide objective evidence for the subjective emotional reports. Although the trends in HRV metrics—specifically the HRV-nLF and HRV-nHF components, which reflect sympathetic and parasympathetic nervous system activity, respectively—did not reach statistical significance, the directional pattern of mean values merits attention. The observed descriptive trend demonstrated a progressive decrease in average physiological arousal levels as participants moved from the red, yellow, and blue to the green workspace. Thus, while not constituting conclusive evidence, the trend in physiological data aligns with findings from previous studies [26,27,28]. For instance, Xie et al. [28] demonstrated that red light elicited the most intense emotional arousal, then blue and green light.

In conclusion, although no dominant emotion words elicited by green were identified in this study, the results demonstrated that the emotional classification of red, yellow, and blue was successfully achieved, indicating the overall success of the emotion classification task.

The study also revealed that the participants’ intrinsic cultural background influenced their positive affect, with Africans reporting higher levels than Asians in the workspace. However, the cultural background and workspace type did not influence their physiological arousal. Additionally, the cultural background × color interaction, workspace type × color interaction, and cultural background × color × workspace type interaction had no significant effects on PA, NA, SCL, HR, SDNN, RMSSD, HRV-nLF, or HRV-nHF.

Furthermore, the results from the IPQ and VRSQ indicated that while the immersive VR experience effectively elicited a strong sense of presence in participants, its adverse effects remained within a controllable range. This combination of high presence and low adverse effects strongly suggests that the virtual workspace possessed high ecological validity. It successfully mirrored the complexities and perceptual qualities of a physical workspace, meaning that the participants’ behaviors and cognitive processes observed within the virtual environment can be reliably generalized to real-word contexts. In addition, the virtual workspace in this study can be a standardized stimulus for emotion induction. Of course, the systematic design framework for constructing immersive virtual reality environments, as proposed in this study, demonstrated transferability and can be replicated and applied in future research.

### Limitations and Recommendations

Although this study successfully established a dataset and revealed fundamental color–emotion associations, several limitations remain.

It must be acknowledged that the application of color schemes in VR is not without potential drawbacks. Firstly, while highly saturated or intense colors can effectively capture attention, prolonged exposure may lead to visual fatigue. Additionally, participants immersed in strong monochromatic environments may experience temporary color adaptation when transitioning between scenes, thereby compromising visual continuity. Secondly, the phenomenon of color adaptation—wherein the perceived impact of colors diminishes with sustained exposure—may reduce their long-term effectiveness. More importantly, although VRSQ results confirmed that the VR workspace environment in this study did not induce significant core cybersickness symptoms (e.g., nausea, dizziness) during the relatively short experimental sessions, the notable incidence of “difficulty focusing” (affecting at least 25% of participants) indicated a risk of visual fatigue. This specific issue could be exacerbated by prolonged usage or more dynamic visual explorations beyond the scope of tasks evaluated in this study. Thus, while the VR environment proved comfortable in terms of cybersickness during short-term exposure, its implications for prolonged visual fatigue warrant further investigation.

Furthermore, the fundamental distinction between color environments generated by VR headsets via light-emitting diodes and real-world settings—where colors result from object reflection of natural or artificial lighting—cannot be overlooked. The complex conditions of physical workspace environments (e.g., variable lighting, spatial layouts, and social interactions) may substantially dilute or alter the psychophysiological effects of colors. Consequently, our findings should be interpreted as providing heuristic insights and hypotheses for real-world design rather than direct solutions. Second, the ecological validity of the present research paradigm requires further direct verification through future “virtual-reality” comparative experiments. Concurrently, the limited statistical power of certain physiological metrics constrains the certainty of their corresponding conclusions. It should also be considered that the generalizability of our findings is limited by the participant sample, which was drawn exclusively from a student population. Therefore, the main directions for future work include conducting specially designed comparative studies, expanding and increasing the diversity of participants, and recruiting individuals from different industries and functional roles. Such research would simultaneously validate the effectiveness of our paradigm and yield more conclusive physiological evidence regarding the impact of color.

In future research, participants will first be asked to select the emotion word that best matches the feelings evoked by green from a set of more specific options. This selection will then be statistically validated to refine the dataset. Ultimately, electroencephalogram (EEG) and other physiological signals will be collected and integrated with machine learning techniques to develop an affective computing model. This model aims to accurately predict the participants’ valence and arousal levels in virtual workspace environments. The current study primarily focused on constructing an affective dataset. Investigating how color directly influences productivity represents an important and valuable direction for future research.

## 6. Conclusions

Through a carefully designed experiment that combined self-reported data (PANAS and arousal) and objective physiological indicators (EDA and HRV), this study accomplished emotion classification for red, yellow, and blue, thereby establishing a dataset for virtual workspace affect. The findings indicated that in the emotional valence, the red workspace significantly induced feelings of “anxious”; the yellow workspace closely elicited individual “happy” mood; the blue space was closely associated with a higher sense of “calm”; while the green space did not exhibit a dominant emotional orientation in this experimental context. In terms of emotional arousal, red and yellow workspaces corresponded to higher arousal states, whereas blue and green led to lower arousal levels. Descriptive trends in HRV-nLF and HRV-nHF also indicated a pattern of decreasing arousal across the red, yellow, blue, and green conditions. The research also found that African participants reported greater positive affect in the workspace compared to Asian participants.

Therefore, this study holds significant theoretical and practical value. Theoretically, given the current scarcity of emotion datasets specifically designed for virtual reality environments, particularly in workspace contexts, the dataset constructed in this study provides a valuable data resource for the field of affective computing. The research findings can be directly applied to and support the development of future “affective intelligence” virtual workspaces, with the potential to enhance the participants’ emotional experiences and overall well-being. Practically, the implications of this study are actionable for architects, interior designers, and organizational managers. The research moves beyond generic color theory to provide evidence-based recommendations for specific functional zones within a workspace. For instance, the data robustly suggest applying warm, energetic hues like yellow in creative collaboration zones. This color was shown to stimulate mental activity and foster a sense of optimism and energy, ideal for brainstorming sessions and team-based innovation. Conversely, utilizing cool tones like blue as the primary color in areas designated for focused. Blue promoted a state of calm and sustained concentration, aiding in tasks that require deep cognitive processing and reducing mental fatigue. Furthermore, the study provides a crucial note of caution, recommending the avoidance of the extensive use of high-saturation red, which was associated with heightened arousal levels that could easily tip into perceived stress or agitation over prolonged periods. This experimental evidence enables a shift away from subjective aesthetic choices toward a model of scientifically informed color planning based on functional zoning. Such an approach enables relative organizations to proactively shape the physical—and virtual—work environment to actively cultivate and optimize participants’ emotional states, thereby supporting not only individual well-being but also enhanced creativity and improved task performance.

## Figures and Tables

**Table 1:** Demographic information of subjects.

Demographic Variable	Value
Total Number	48
Male	16
Female	32
Age Range	18–34 years
Asian (Chinese, Bangladeshi, Indonesian)	36
African (Congolese, Ethiopian, Ugandan, Gambian, Nigerien, Nigerian, Rwandan)	12

**Table 2:** Comparison of each label in the condition with the highest rating and all other conditions.

VS	Red	Yellow	Blue	Green
Stimulus Color/Emotion Label	Estimate (*p*)	Estimate (*p*)	Estimate (*p*)	Estimate (*p*)
Red/Anxious		102.257 (<0.001)	63.431 (0.003)	54.632 (<0.001)
Yellow/Happy	80.889 (<0.001)		79.083 (<0.001)	81.056 (<0.001)
Blue/Calm	92.785 (<0.001)	−11.743 (0.513)		44.569 (0.028)

Note: Estimate represents the differences in aligned rank-transformed means. *p* values are adjusted using the Tukey HSD method.

**Table 3:** Mean and standard deviation (Mean ± SD) of all participants’ ratings for each color.

Color	Arousal	Label
Red	2.896 ± 1.072	High arousal
Yellow	2.667 ± 1.209	High arousal
Blue	2.365 ± 1.279	Low arousal
Green	2.365 ± 1.090	Low arousal

## Data Availability

The data that support the findings of this study are available from the corresponding authors.
